# A shared representation of order between encoding and recognition in visual short-term memory

**DOI:** 10.1016/j.neuroimage.2017.04.047

**Published:** 2017-07-15

**Authors:** Kristjan Kalm, Dennis Norris

**Affiliations:** Cognition and Brain Sciences Unit, Medical Research Council, 15 Chaucer Road, Cambridge CB2 7EF, UK

**Keywords:** Short term memory, Sequence position, Positional code, Temporal position, fMRI, Neuroimaging

## Abstract

Many complex tasks require people to bind individual events into a sequence that can be held in short term memory (STM). For this purpose information about the order of the individual events in the sequence needs to be maintained in an active and accessible form in STM over a period of few seconds. Here we investigated how the temporal order information is shared between the presentation and response phases of an STM task. We trained a classification algorithm on the fMRI activity patterns from the presentation phase of the STM task to predict the order of the items during the subsequent recognition phase. While voxels in a number of brain regions represented positional information during either presentation and recognition phases, only voxels in the lateral prefrontal cortex (PFC) and the anterior temporal lobe (ATL) represented position consistently across task phases. A shared positional code in the ATL might reflect verbal recoding of visual sequences to facilitate the maintenance of order information over several seconds.

## Highlights

• Representing sequences in a short-term memory (STM) task requires a shared positional code between encoding and recall.
• We present first multivariate analysis of imaging data from a STM task of sequence position representation.
• Most of sensory and association cortices represented positional code within a single task phase.
• Only lateral prefrontal cortex and the anterior temporal lobe represented position consistently across task phases.

## Introduction

**Fig. 1:**
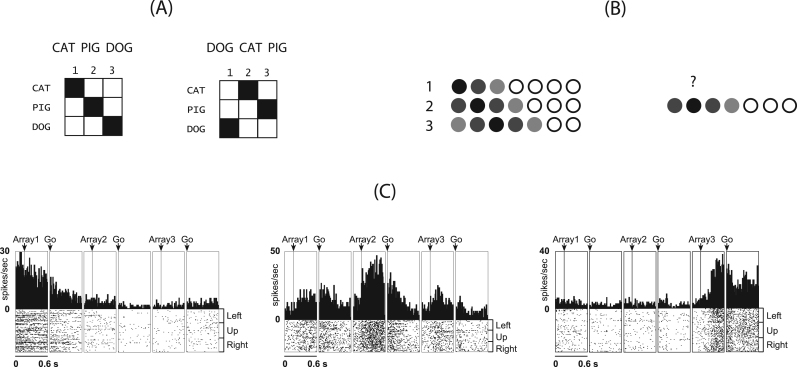
Sequence representation and positional code. (A) Representation of two sequences as mappings between item codes and temporal position codes. (B) Left: representation of the temporal position of three first items in a 7-item sequence. The variance around positional code is represented in terms of the darkness of the circle. Right: the item at position two is retrieved by reinstating each positional code which then cues the associated item. (C) Examples of temporal position selective neurons from Berdyyeva and Olson (2010). From left to right: Pre-supplementary motor area neuron selective for 1st position, Supplementary eye field neuron selective for 2nd position, and Supplementary motor area neuron selective for the 3rd position in the serial object task.

One of the most important features of human short term memory (STM) is the ability to bind individual events into a sequence. A host of complex behaviours including language processing, vocabulary acquisition, and chunk formation are thought to rely on sequence encoding in STM (see Hurlstone et al. (2014), for a review). Information about the order of the individual stimuli in the sequence needs to be held in an active and accessible form in STM over a period of few seconds (Botvinick and Watanabe, 2007, Baddeley, 2003). Research has shown that the position of a stimulus in a sequence is encoded in STM separately and independently of its identity (Henson and Burgess, 1997, Henson et al., 1996, Page and Norris, 2009, Fig. 1A). From hereon we refer to such neural representation of an item's position in the sequence as *positional code*. Fig. 1C gives an example of a simple positional code showing the responses of position-sensitive neurons from monkey Supplementary motor area, as observed by Berdyyeva and Olson (2010).

The neural implementation of the positional code has been extensively studied in animal neurophysiology. Neurons selective for each position in a sequence have been observed in monkey dorsolateral prefrontal cortex (Averbeck and Lee, 2007, Inoue and Mikami, 2006, Ninokura et al., 2004, Barone and Joseph, 1989), Supplementary and Presupplementary motor area (Nakajima et al., 2009, Berdyyeva and Olson, 2010, Isoda and Tanji, 2004), and medial premotor cortex (Crowe et al., 2014, Merchant et al., 2013). Other research on animal neurophysiology and human neuroimaging has suggested that the hippocampus encodes the position of items in a sequence (Heusser et al., 2016; Rangel et al., 2014; Hsieh et al., 2014; DuBrow and Davachi, 2014; Ginther et al., 2011), with some authors proposing the existence of’time cells’ tracking the temporal information during sequence processing (MacDonald et al., 2011, MacDonald et al., 2013).

In the current paper we investigate how the positional code is represented in human STM. By contrast, previous human neuroimaging studies have focussed on the representations elicited by learned sequences (Ross et al., 2009, Albouy et al., 2008, Schendan et al., 2003, Hsieh and Ranganath, 2015, Hsieh et al., 2014), which can be assumed to be represented very differently from those maintained in STM. Similarly, in many studies the task has not required participants to actively retain the order of the stimuli in memory (Heusser et al., 2016, DuBrow and Davachi, 2016, DuBrow and Davachi, 2014, Amiez and Petrides, 2007, Hsieh and Ranganath, 2015, Hsieh et al., 2014). No previous imaging studies have reported multivariate analyses of an order STM task. Furthermore, previous studies have not addressed the fact that several unrelated cognitive processes, such as memory load, sensory adaptation, and reward expectation, also change in a consistent manner as the sequence unfolds. Therefore it becomes difficult to ascertain whether their results are actually indicative or order memory or a collinear change in some other variable such as memory load (for a detailed treatment of this issue see Kalm and Norris (2016)).

Here we used an STM task where participants had to remember and subsequently recognise a short sequence of images. In order to recall items in the correct order participants had to retrieve the positional code instantiated during the presentation phase of the STM task (Fig. 1B). We investigated whether any brain regions shared this positional code between the presentation and response phases of the task. For this purpose, we trained a classification algorithm to use the fMRI activity patterns of individual items to predict the positions of those items when they appeared in different sequences. Such an analysis abstracts over item identity (such as a specific image) but is sensitive to the representations that consistently code for an item's position within a sequence. Importantly, we used activity patterns from the presentation phase of the STM task to predict the position of the items during the subsequent recognition phase. This allows us to identify brain regions where the positional code is shared between encoding and response phases. Representations shared by encoding and recognition should reflect a common memory representation of order and not other sequential processes such as memory load or sensory adaptation. In sum, we consider our study to be the first controlled fMRI experiment to study the order representations in STM.

Our results revealed that although several brain regions showed sensitivity to order within a single phase, only the voxels in the lateral prefrontal cortex (PFC) and the anterior temporal lobe (ATL) represented item position consistently across task phases. This suggests that while many brain areas, including sensory and motor cortices, are sensitive to temporal position, those representations might not be used to guide behaviour and could instead reflect perceptual or load-related aspects of the task. Our findings suggest that voxels in the PFC and ATL are not only sensitive to sequentially presented stimuli (Amiez and Petrides, 2007) or sequentially executed actions (Averbeck and Lee, 2007) but encode temporal position information across task phases in a manner which could be used to guide behaviour.

## Methods

### Participants

In total, 13 right-handed volunteers (6 female, 20–33 years old) gave informed, written consent for participation in the study after its nature had been explained to them. Subjects reported no history of psychiatric or neurological disorders and no current use of any psychoactive medications. Two participants were later excluded from the study because of excessive motion artefacts in the collected fMRI data (see *Physiological noise removal* for the exclusion criteria). The study was approved by the Cambridge Local Research Ethics Committee (LREC) (Cambridge, UK).

### Task

**Fig. 2:**
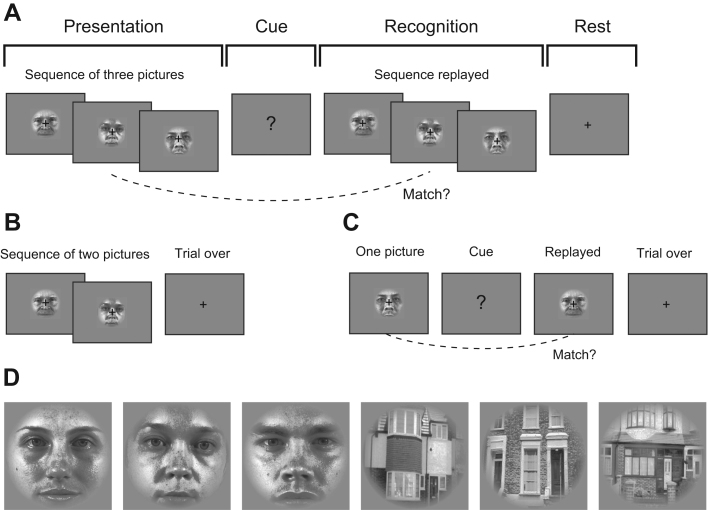
Examples of a single trial: (A) Three-item sequence where all items were presented in the recognition phase, item-order mappings remained the same; (B) two-item sequence without recognition, (C) single item’sequence’ with recognition, item-order mapping not the same. (D) Examples of stimuli.

We used an immediate serial recognition task where participants had to remember sequences of one, two or three pictures of houses or faces in the order they were presented (Fig. 2). On each trial participants were presented with a visual fixation cross to indicate the start of the presentation of the sequence. During the presentation phase, pictures of houses or faces were presented individually (each item for 3.5 s so as to obtain two scans per item) followed by a brief delay (2 s). This was followed by an order recognition phase, where a replay of the initial sequence was displayed. At the end of this phase participants had to indicate whether the items in the sequence were presented in the same order as in the original sequence (Fig. 2). In order to ensure that participants paid constant attention during recognition we changed the replayed sequence on 8 trials out of 96. Items which did not appear in their original presented positions during those trials were not included in the later fMRI data analysis. On 1/3 of the trials the recognition phase (replay of the sequence) was omitted. The recognition phase was followed by a cue+indicating that there would be a delay of between 6 and 16 s before the next trial. The inclusion of the recognition phase in the trial was randomised across the experiment.

We used short 3-item sequences to ensure that the entire sequence could be accurately retained in STM. If we had used longer sequences then the representation of any given sequence would necessarily vary from trial to trial depending on the nature of the errors, and no consistent pattern of neural activity could be detected. Furthermore, we wanted to estimate separate regressors for individual items in the sequence during both the presentation and recognition phases of the tasks. Presenting stimuli sequentially in an event related fMRI design poses substantial problems for later data analysis: using temporally adjacent stimuli without an intervening rest period creates collinearity in the fMRI data due to the temporal lag of the haemodynamic response function. This in turn makes it difficult to estimate BOLD responses separately for the individual items in the sequence. We took a number of steps to address this issue in the task design. First, the number of items in the sequence was varied randomly across the trials. This ensured that the first item in the sequence was not always followed by a second item, and similarly the second item not by a third. As a result, 44% of the presented sequences were three items long, 39% were two items long and 17% one item long (see Table 1). Second, participants’ memory was probed only on 2/3 of the trials so that we could model the fMRI responses from presentation and response phases separately. Each participant was presented with 96 trials in a single scanning run in addition to an initial practice session outside the scanner. Participants were not informed that there were different types of trials.

**Table 1:** Structure of trials and items presented during the experiment.

A - Types of trials			
		*Trial phase*	
	Sequence length in items	Presentation	Recognition
	3	22	15
Houses	2	18	12
	1	8	16
			
	3	22	15
Faces	2	18	12
	1	8	16
			
Total		96	66

B - Types of items in sequences			
		*Trial phase*	
	Position in sequence	Presentation	Recognition
	1	48	32
Houses	2	40	27
	3	22	15
			
	1	48	32
Faces	2	40	27
	3	22	15
			
Total		220	148

### Stimuli

We used three individual images of houses and faces (six different images in total as shown on Fig. 2D). All presented sequences were permutations of the same three images of houses or faces (from hereon called items). To ensure that the behavioural task measured order memory alone all items appeared at all sequence positions and there was no novel item-based information in any sequence. It was impossible to present all items equally at all positions since we used one-, two- and three-item sequences. However, all items appeared at different positions as equally as possible: the distribution of items at different sequence positions is given in Table 1.

The images of faces and houses were processed in Matlab to achieve similar luminance histograms, and were cropped to ensure that each image appeared in a similar retinal area. Cropping was achieved with a smooth border, and the resulting image was superimposed on a grey background (Fig. 2). The stimuli subtended a 6° visual angle around the fixation point in order to elicit an approximately foveal retinotopic representation. Stimuli were back-projected onto a screen in the scanner which participants viewed via a tilted mirror. The experiment was controlled using Matlab and the Psychophysics Toolbox extension (Kleiner et al., 2007).

### fMRI data acquisition and pre-processing

Participants were scanned at the Medical Research Council Cognition and Brain Sciences Unit (Cambridge, UK) on a 3 T Siemens TIM Trio MRI scanner using a 32-channel head coil. Functional images were collected using 32 slices covering the whole brain (slice thickness 2mm, 25% slice gap, in plane resolution 2×2 mm) with *TR*=1.75 s and *TE*=44 ms. In addition, MPRAGE structural images were acquired at 1mm isotropic resolution. (See http://imaging.mrc-cbu.cam.ac.uk/imaging/ImagingSequences for detailed information.) All volumes were collected in a single, continuous run for each participant. 756 volumes were acquired in a single acquisition run which lasted approximately 22 min. The initial six volumes from the run were discarded to allow for T1 equilibration effects. All fMRI data were pre-processed using SPM8 software (Wellcome Trust Centre for Neuroimaging, London) and analysed using custom in-house software. Prior to analysis, all images were corrected for slice timing, with the middle slice in each scan used as a reference. Images were realigned with respect to the first image using tri-linear interpolation, creating a mean realigned image. The mean realigned image was then co-registered with the structural image and the structural image was normalized to the MNI average brain using the combined segmentation/normalization procedure in SPM8. The functional volumes remained unsmoothed and in their native space for participant-specific generalized linear modelling.

### Physiological noise removal

In order to remove physiological noise from the fMRI signal we measured respiratory and cardiac data during scanning and used them as nuisance regressors in the general linear model (GLM). A pulse oximeter was used to record participants’ cardiac data, and a pneumatic breathing belt to record the respiratory data. The physiological data were then filtered and down-sampled using the PhLEM toolbox (Verstynen and Deshpande, 2011) to match the scan acquisition time and added to the GLM as separate nuisance regressors. Six motion parameters corresponding to translations and rotations of the image due to movement in the scanner, and additional scan-specific regressors were also added to account for large head movements. Additional parameters were modelled to account for extreme inter-scan movements which exceeded a translation threshold of 0.5 mm, rotation threshold of 1.33° and between-images difference threshold of 0.035 calculated by dividing the summed squared difference of consecutive images by the squared global mean. Two participants were excluded from the study because more than 10% of the acquired volumes had extreme inter-scan movements.

### General linear model and event regressors

#### Event regressors

We sought to dissociate fMRI activity patterns representing the identity of the items from the patterns representing their position within the sequence. As noted above, when stimuli are presented in immediate succession without an intervening rest period this creates collinearity in the fMRI data due to the temporal lag of the HRF. We took a number of steps to address this issue in the experiment design (see also *Task* above). First, we randomised the number of items in the sequence and their order of appearance across the trials. Second, we presented each item for 3.5 s to obtain two scans of data per item in the sequence. Third, we omitted the response phase of the task on approximately 1/3 of the trials. Fourth, we jittered the duration of the rest phase between 6–16 s. Fifth, no temporal decorrelation or whitening of fMRI data was carried out at task-relevant frequencies prior to estimating the general linear model (GLM) to avoid artificial dissimilarities between adjacent events. Finally, every event regressor was estimated with a separate GLM to avoid an overlap in temporally adjacent regressor estimates. This process included combining individual events so that the number of final regressors was balanced across event types (see Table 2).

**Table 2:** Distribution of fMRI data regressors for every participant.

		*Trial phase*	
	Position in sequence	Presentation	Recognition
	1	14	9
Houses	2	14	9
	3	14	9
			
	1	14	9
Faces	2	14	9
	3	14	9
			
Total		42	27

As a result of these measures we obtained a sufficient degree of decorrelation between the event regressors in the GLM for every position in the sequence. Finally, nuisance regressors were added to each GLM modelling head movement and cardiac and respiratory activity (see *Physiological noise removal*).

To further ensure decorrelation we combined the same types of events across the experiment (see Table 1B for the exact event label combinations) into single regressors. To obtain balanced training sets for the subsequent classification analysis we estimated an equal number of event regressors within a task phase (presentation or recognition). This was done by randomly selecting *n* occurrences of a unique combination of event labels: item set (houses or faces), item identity (one of three houses or faces), position in a sequence (first, second, or third), and task phase (presentation or recognition) and assigning them a unique regressor label. The instances of the same event type were selected a pseudo-randomly determined distance apart over the course of the experiment to average out the effects of temporal proximity in the fMRI data. For example, a recognition phase event label which was presented 27 times during the experiment (e.g. second item in a 3-item sequence of houses, Table 1B) was combined into 27*/*3=9 event regressors for the fMRI analysis so that each resulting regressor averaged over *n*=3 individual occurrences of that event type. Presentation phase events were combined into 14 event regressors and recognition phase events into 9 regressors (Table 2). Where the number of event labels was not the integer of *n* the last regressor contained the remainder of the events (2 or 1).

#### GLM estimation

The event regressors were convolved with the canonical haemodynamic response (as defined by SPM8 analysis package) and passed through a high-pass filter (128 s) to remove low-frequency noise. Parameter estimates (beta weights) measuring the brain activity evoked during each event type were estimated with the Least-Square2 method (Turner et al., 2012) so that a separate GLM was estimated for each beta. This ensured that the overlap between adjacent event regressors did not affect the estimated beta weights. The resulting beta volumes were grey-matter-masked using the tissue probability maps generated by the segmentation processing stage and were used as inputs for multi-voxel pattern analysis. As a result, we obtained 69 images representing the individual sequence items from both presentation and recognition phases of the experiment (see Table 2) for each participant.

### Multi-voxel pattern analysis

A number of methodological issues need to be addressed when performing an fMRI experiment where the aim is to investigate the representation of temporal position. The central problem is that items in different positions necessarily differ along other dimensions too. An item in position three is preceded by more items than one in position two and occurs at a later time than item two. In a memory task, memory load will be greater at position three than position two. Any or all of these factors might lead to an increase or decrease in activation over position, and this would provide the information necessary for a linear classifier to discriminate between items in different positions. These challenges and how they can be overcome are covered in detail by Kalm and Norris (2016). To briefly summarise, we used two methods to ensure that classification was based on the positional code rather than information collinear to the positional information. First, we excluded univariate changes between sequence items by z-scoring the activation of all voxels with respect to their mean activity before the analysis. This ensured that our classification analysis was insensitive to changes which affect a brain region uniformly, such as sensory adaptation or memory load. Second, we employed an analysis where training and testing data came from two different task phases. This ensured that the accuracy of the classification was based on the information shared by two different behavioural stages and hence any effects of the particular task phase (presentation or recognition) would be cancelled out. We refer the reader to Kalm and Norris (2016) for a detailed account of these issues.

#### Classification analysis

We moved a spherical searchlight with a 6-mm radius throughout the grey-matter masked and unsmoothed volumes to select, at each location, a local contiguous set of 124 voxels (2 mm isotropic). As a result, the voxels in the searchlight comprised a vector of activations from beta images resulting in 124×69 matrix of *voxels*×*sequence items*. Voxel vectors where then z-scored to exclude any univariate effects from the analysis.

To identify voxels which encoded position information we ran a 3-way classification of item position in a sequence for both stimulus types (houses and faces). We labelled the voxel vectors according to their position in the sequence (1, 2, or 3; see *Stimuli* for details) and split the vectors into two data sets: a training set used to train a support vector machine (SVM, with a linear kernel and a regularization hyper-parameter *C*=40) to assign correct labels to the activation patterns (1, 2 or 3), and a test set (including one sample from each class) used to independently test the classification performance. The SVM classifier was trained to discriminate between the three order positions with the training data, and subsequently tested on the independent test data.

We carried out three classification analyses: position classification during the presentation and recognition phases, and a cross-task phase position classification. In the first two both training and testing data came from the same phase of the task (presentation or recognition). We used leave-3-out cross-validation (one test item per each of 3 classes) to obtain a mean classification accuracy for a searchlight and then calculated an estimate of a true classification accuracy (see *Significance testing* below). In the cross-phase analysis the training data came from the presentation phase of the trial while the testing data came from the recognition phase of the trial. Here cross-validation was a-priori provided by two task phases. The classification was performed with the LIBSVM (Chang and Lin, 2011) implementation.

For every participant, the classification searchlight analysis resulted in a classification accuracy brain map. We assigned a score of zero to any sphere in which fewer than 33 voxels were inside the individual grey matter volume. These individual images were subsequently normalised to the MNI anatomical template and entered into group-level analyses.

#### Significance testing

We assessed classification accuracies statistically with non-parametric randomization tests (Stelzer et al., 2012). We permuted the correspondence between the test labels and data 100 different times to compute 100 mean classification accuracies for the testing labels. To this permuted distribution of accuracies we added the mean accuracy obtained with the correct labelling. We then obtained the distribution of group-level mean accuracies by randomly sampling 1000 mean accuracies (with replacement) from each participant's permuted distribution. Next, we found the true group-level mean accuracy's empirical probability based on its place in a rank ordering of this distribution. The peak percentiles of significance (*p*<0.001) are limited by the number of samples producing the randomized probability distribution at the group level.

## Results

### Behavioural results

As would be expected given that the sequences were no more than three items long, all participants performed at or very near ceiling: the average number of incorrect recognition decisions was 0.6 out of 96 trials (99.4% mean accuracy). The data from incorrectly recalled trials were excluded from the imaging analysis (see *Methods*, *Event regressors*).

### Representation of the position of individual items in STM

**Fig. 3:**
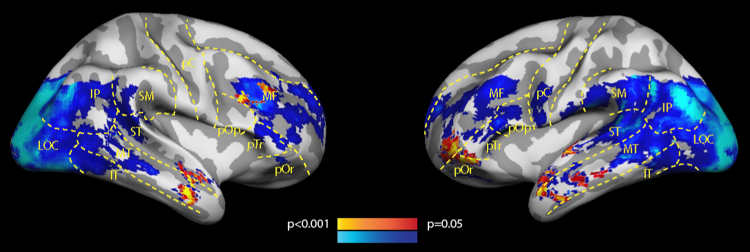
Regions where the position of the items within a sequence was decoded significantly above chance across participants: red-yellow – significantly above chance between task phases, blue-cyan – significantly above chance within a presentation task phase only. Note that within-phase and across-phase classification maps are not directly comparable and are overlaid here only for visualisation purposes (see Supplementary Information ”Item position classification accuracy within single task phases” for more information). Abbreviations correspond to the following cortices: MF – middle frontal lobe, pOr – pars orbitalis, pTr – pars triangularis, pOp – pars opercularis, pC – precentral area, ST – superior temporal lobe, MT – middle temporal lobe, IT – inferior temporal lobe, LOC – lateral occipital lobe, SM – supramarginal area.

We ran a whole-brain searchlight classification analysis to identify which brain regions shared the positional code between the presentation and recognition phases of the task. Classification was significantly above chance bilaterally in the rostro-lateral prefrontal cortex (rlPFC) and anterior portion of the superior and middle temporal lobe (Fig. 3 – red-yellow accuracy map). There was no significant difference in decoding accuracy between different classes of stimuli (houses and faces, *df*=12, *p*=0.63). When classification was carried out within a single task phase only (presentation or recognition) we could decode item position in the sequence within large portions of the lateral occipital cortex and posterior portions of the temporal lobes (Fig. 3 – blue-cyan accuracy map).

**Fig. 4:**
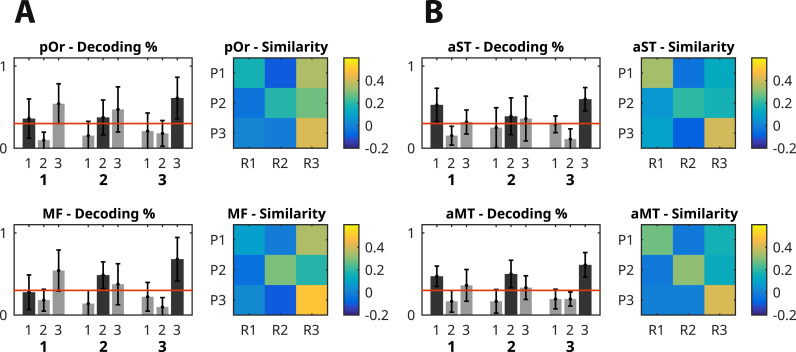
Classification accuracy and pattern similarity between two task phases in rostro-lateral prefrontal (A) and anterior temporal regions (B). Bar charts display the average classification accuracy across participants by comparing the known position labels (bar groups) to the predictions made by the classification algorithm (bars within the group). Bars show the proportion of predicted values for each position. Correct classifications are represented with a darker bar. Error bars show the standard error of the mean. The red line depicts the chance level classification accuracy 1/3. Similarity matrices display average pairwise pattern correlations (Pearson's *ρ*) between two task phases: P – presentation, R – recognition, 1, 2, 3 – position. Cells on the diagonal show the pattern correlation within the same positions between two task phases. Abbreviations: MF – middle frontal lobe, pOr – pars orbitalis, aST – antero-superior temporal lobe, aMT – anterior middle temporal lobe.

Next we investigated whether above-chance decoding across task phases was based on similar patterns across different brain areas. For this purpose we carried out pattern similarity analysis in above-chance voxel clusters in anatomically distinct brain regions (defined by the Desikan-Killany atlas; Desikan et al., 2006). The results of pattern similarity analysis revealed differences in the way antero-temporal and prefrontal cortices represent positional code across task phases. We observed that in the rlPFC regions the first position in the sequence was significantly misclassified compared to the second or the third positions. This can be directly observed by comparing the known positions of the items to the predictions made by the classification algorithm (Fig. 4A, left column). In one of the rlPFC regions (*pars orbitalis*) items in any position (1st, 2nd, or 3rd) were likely to be classified as 3rd position items, and all items had high similarity to the patterns of 3rd position items. Contrastingly, in the anterior temporal lobe (ATL) regions all positions were on the average classified correctly (Fig. 4B, left column).

**Fig. 5:**
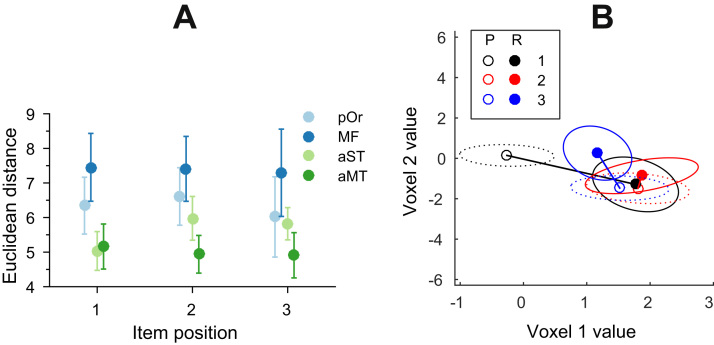
(A) Change in position representations across task phases averaged over participants in prefrontal (blue) and temporal (green) regions of interest. Error bars show the standard error of the mean. Analysis of variance was significant for region (*F*=10.38, *df*=3, *p*<0.001) but not position (*F*=0.23, *df*=2, *p*=0.79). (B) Change in position representations for a single subject between two task phases. For visualisation purposes only values from two most discriminant voxels are plotted. Empty markers represent the distribution of means for the presentation phase, filled markers for the recognition phase. Black – first position, red – second position, blue – third position. Ellipses represent two standard deviations around the mean: dotted ellipses – presentation, solid ellipses – recognition. Straight lines represent the Euclidean distance the mean of the distribution has moved in two-voxel space between presentation and recognition.

These results suggest that in the rlPFC the representation of the position changed significantly between task phases compared to the temporal lobe ROIs. Analysis of variance on how much the distribution mean for each position representation had moved between task phases (with respect to the decision boundary of the classifier) showed a significant effect of region (*F*=10.38, *df*=3, *p*<0.001) but not position (*F*=0.23, *df*=2, *p*=0.79) or interaction (*F*=0.33, *df*=6, *p*=0.92). Fig. 5A shows change in position representation across task phases for both prefrontal and temporal ROIs.

The difference in mean position-wise distances between task phases is unlikely to be due to different noise profiles as there was no significant effect of variance across or within task phases in terms of item position (*df*=12, *p*=0.31). Fig. 5B illustrates this representational change with a single subject example: here the activation distribution for the first position changed significantly between task phases while the distributions for the second and third positions changed less.

In sum, although we could predict the position of individual items in several brain areas, only regions in the rlPFC and ATL encode position across task phases. However, out of the two regions, only in the ATL the representations for all three sequence positions are consistent over the duration of the STM task: pattern similarity analysis revealed in the rlPFC regions representations for the first positions change so that they could not be reliably decoded during the recognition phase.

## Discussion

In this paper we investigated whether any brain regions showed evidence of a positional code that was common to both the presentation and response phases of the STM task. We found that while voxels in a number of brain regions represented positional information during either presentation and recognition phases, only voxels in the rostro-lateral prefrontal cortex (rlPFC) and the anterior temporal lobe (ATL) represented position consistently across task phases.

This suggests that a positional’read-out’ in the sensory cortices (Fig. 3 – blue-cyan accuracy map) is not indicative of STM representations of the positional code but rather reflects perceptual or load-related aspects of the task. Our findings suggest that only position representations in the PFC and ATL could encode temporal position information across task phases (Fig. 3 – red-yellow). Furthermore, only in the ATL were all three sequence position representations shared between task phases.

### The shared positional code in the lateral ATL

Regions in the lateral ATL have been previously shown to serve linguistic and auditory processing (Visser et al., 2010), including semantic features of the stimuli (for a review see Bonner and Price (2013)). This raises a question about the nature of the positional code in the ATL as we used *visual* stimuli in our STM task. Hence, our findings suggest that participants could engage in auditory-verbal recoding of unfamiliar visual stimuli. Numerous studies have observed verbal recoding of visual stimuli in STM tasks (bino, 1993, Palmer, 2000, Brandimonte et al., 1992). Furthermore, visual sequences have been shown to be encoded differently from auditory sequences, leading to qualitatively different serial position curves (Hurlstone et al., 2014, Crowder, 1986, Conway and Christiansen, 2005). Saffran (2003) showed that repeated presentations of an item in the same position improved learning for auditory stimuli, and for simultaneously presented visual stimuli, but not for sequentially presented visual stimuli. Hence our data, together with previous findings, suggest that unfamiliar visual sequences might be recoded verbally to facilitate the maintenance of positional codes between STM task phases. In other words, as the information about the order of the individual stimuli in the sequence needs to be in active and accessible form in STM over a period of few seconds, verbal recoding and rehearsal might help to retain the positional code between initial instantiation and subsequent recall.

### The positional code in the PFC is not stable across task phases

Our results replicate previous studies which have observed neural positional code in the lateral prefrontal cortices of monkeys (Averbeck and Lee, 2007, Inoue and Mikami, 2006, Ninokura et al., 2004, Barone and Joseph, 1989) and humans. Amiez and Petrides (2007) found that human mid-dorsolateral PFC areas 46 and 9/46 were more activated during the presentation phase of temporal order task compared to the control task of stimulus identification. However, animal studies have almost exclusively used motor tasks to probe temporal order memory by requiring the animal to recreate some aspect of the sequence through motor responses (for a review see Kalm and Norris (2016)). As noted by Averbeck and Lee (2007), this motor component of the task makes it hard to distinguish between sequential action planning and item-independent memory representations of position. However, unlike in the ATL regions, not all position representations were shared between the presentation and recognition phases. Specifically, we observed the first position representation changed significantly after the presentation so that it was consistently misclassified during the recognition phase (Fig. 4). The analysis of pattern similarity in PFC regions revealed that individual order representations were much further apart during the presentation phase compared to the subsequent recognition phase when they became more clustered together (Fig. 5). This caused significant misclassification in the PFC regions since our classification algorithm used presentation data to predict recognition phase labels. This suggests that the positional code in the PFC is either susceptible to processes which evolve along the sequence but do not represent position (such as memory load or sensory adaptation) or represent the start of the sequence in some specific way. Neurons in the PFC have been observed to change their response patterns both in terms which stimulus features they respond to and the amplitude of the responses within a single task and experimental session (Barone and Joseph, 1989, Ninokura et al., 2004, Berdyyeva and Olson, 2011).

### The positional code in the STM task

Our study is the first to examine the position of individual items with fMRI in a STM task requiring memory for order. Previously, Hsieh and Ranganath (2015) carried out an fMRI study focussing on the representation of learned sequences (Hsieh and Ranganath, 2015, Hsieh et al., 2014). The authors also presented’unlearned’ sequences and analysed the data from those trials in terms of the pattern similarity of the position of individual items. They found that the voxels in the parahippocampal cortex encoded the presented objects in terms of position (Hsieh and Ranganath, 2015, Hsieh et al., 2014). However, the study did not investigate STM for order as participants were not required to retain or recall order information whilst being scanned, but instead had to make a semantic judgement on each visually presented object. As a result, it is difficult to suggest that the observed differences in pattern similarities in random sequences were attributable to the representation of a positional code in STM. Contrastingly, here we used a task where participants actively encoded, maintained and recalled an equal number of random sequences within their STM span. This ensured that the position information in each sequence were not yet learned and the representations had to be stored in STM.

Results from physiology and imaging studies in animals and humans indicate that the medio-temporal lobe (MTL) plays a critical role in sequence memory. A large body of evidence suggests that the hippocampus proper encodes the associations between individual items and their positions (Manns et al., 2007, Devito and Eichenbaum, 2011, Hsieh et al., 2014, Hsieh and Ranganath, 2015, Heusser et al., 2016, Naya and Suzuki, 2011). When the hippocampus is pharmacologically inactivated, rodents lose the ability to remember the sequential ordering of a series of odours (Devito and Eichenbaum, 2011, Kesner et al., 2010, Fortin et al., 2002). However, a common feature of these studies is that they have examined the representation of learned sequences. In contrast, we examined the representation of unfamiliar sequences which had to be temporarily maintained in STM.

### Conclusions

Our results reveal that only the voxels in the lateral prefrontal cortex and the anterior temporal lobe represented item position consistently across visual STM task phases. This suggests that while many brain areas, including sensory and association cortices, are sensitive to temporal position, those representations might not be used to guide behaviour and could instead reflect perceptual or load-related aspects of the task. We suggest that shared positional code in the temporal lobe might reflect verbal recoding of visual sequences to facilitate the maintenance of order information over several seconds.
