# Supplementary material for: A shared representation of order between encoding and recognition in visual short-term memory
Source: Neuroimage. 2017 Jul 15;155:138–46. doi: 10.1016/j.neuroimage.2017.04.047 (PMC5518770; doi:10.1016/j.neuroimage.2017.04.047)
Supplement: Supplementary material [file mmc1.pdf]

Supplementary information

Distribution of behavioural errors across participants

Figure 1: Distribution of behavioural errors across participants

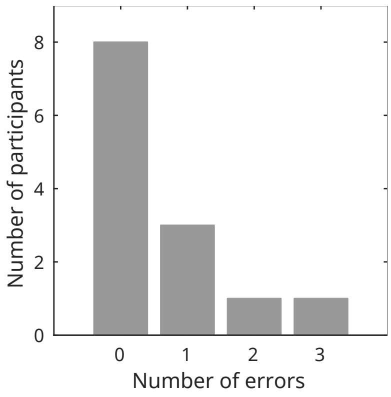

Classification accuracy in the anatomically defined regions of interest in the medio-temporal lobe

Besides searchlight analysis we ran position classification analysis across task phases in several functional areas in ventral visual processing streams. Bilateral ROIs corresponding to the hippocampus (HC) and the parahippocampal cortex (PHC) were defined for each participant using criteria described in previous studies (Insausti et al., 1998). The PHC was defined as the cortex on both banks of the collateral sulcus. The rostral limit of the PHC was defined 2 mm anterior to the limen insula. The caudal limit of the PHC was defined as the caudal limit of the HC. The rostral limit of the HC was defined as the border with the amygdala and the caudal limit was defined as the border with white matter and the tail of the lateral ventricle. The bilateral ROIs for both regions were also divided into separate left and right hemisphere ROIs in order to determine whether there were any qualitative differences between the two hemispheres. The classification accuracy was not significantly above chance ($p < 0.05$) in any of the anatomically defined regions of interest.

Item position classification accuracy within single task phases

Note that within-phase classification maps are not directly comparable to the across-phase classification map because of the differences in the classification mechanism. As described in the Methods section, within-phase classification used leave-3-out cross-validation runs over 27 data points (Table 2) to calculate an average classification accuracy for a given searchlight. This lead

to $9^3 = 729$ cross-validation runs per searchlight. Such cross-validation is impossible for across-phase classification where only a single run per searchlight can be performed (training data from presentation phase and testing data from recognition phase). Therefore null-distributions for within-phase and across-phase classifications are different for the same searchlight. However, within-phase maps still provide a useful visualisation of the extent of the cortex where item decoding is possible when not controlled for collinear variables.

Figure 2: Presentation phase

(A) Lateral

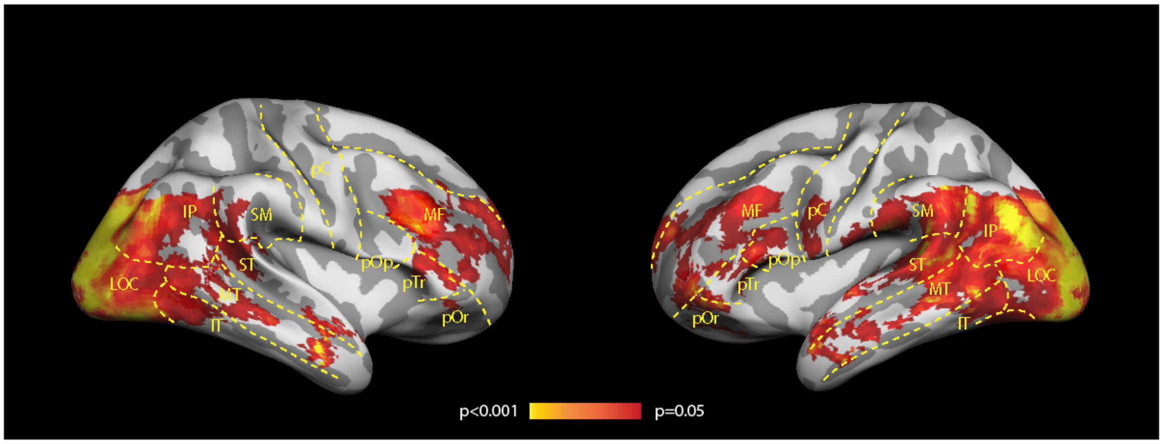

(B) Medial

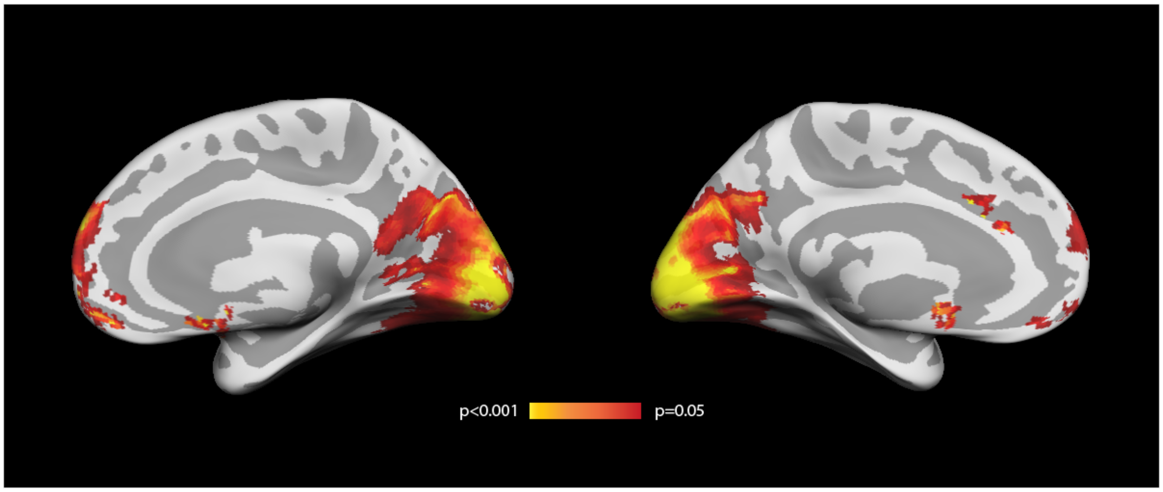

(C) Ventral

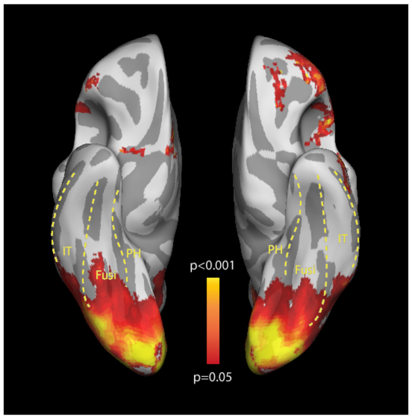

Figure 3: Recognition phase

(A) Lateral

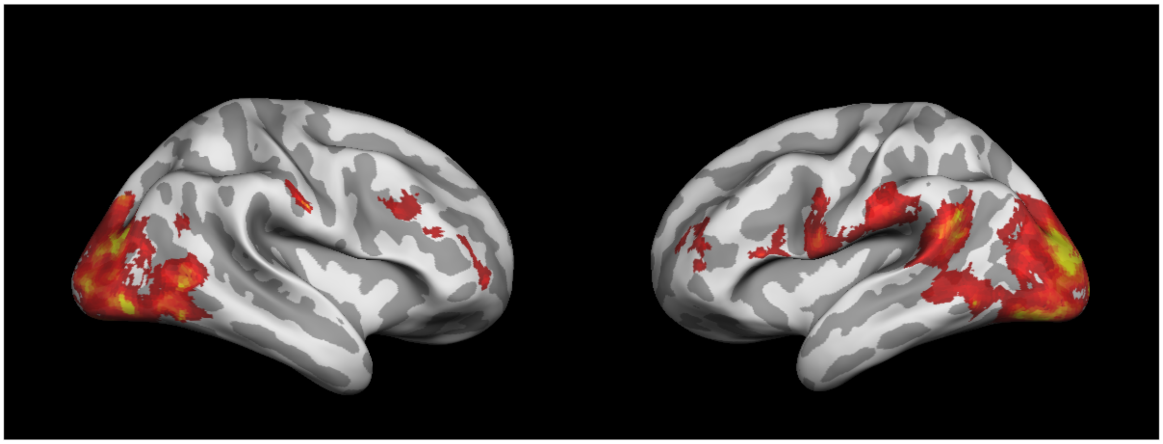

(B) Medial

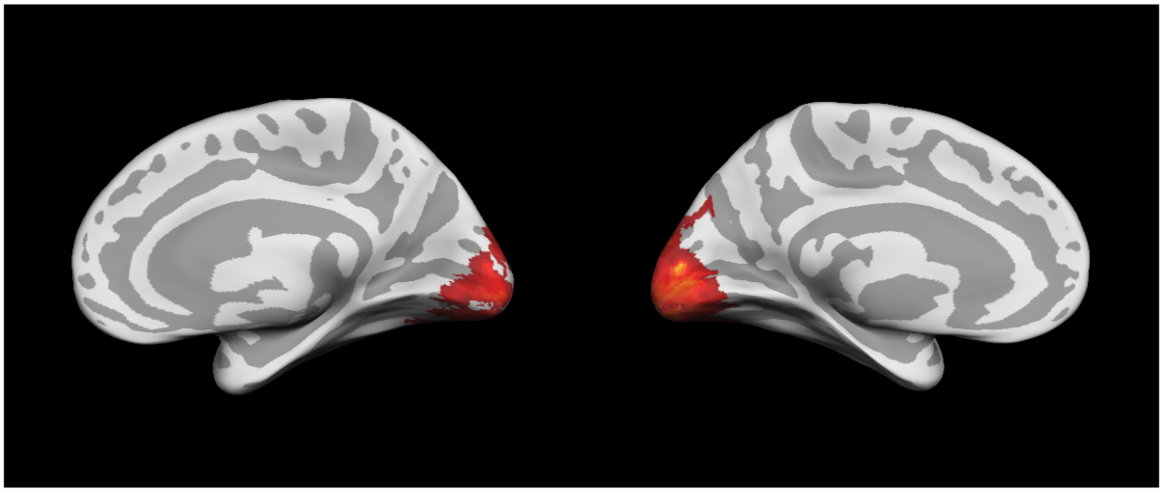

(C) Ventral

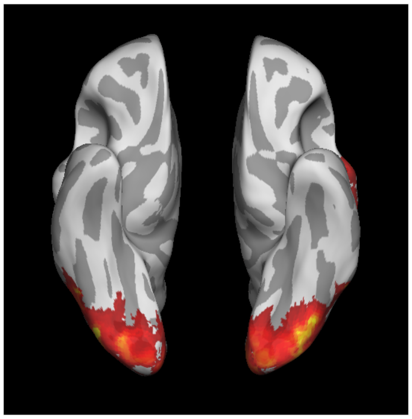

Tables of peak clusters

Table 1: Presentation phase

Size(n)	MNI X	MNI Y	MNI Z	Maximum t-value
22	36	22	30	29.12
4518	-2	-94	-4	24.41
383	-44	-76	24	21.92
122	26	-74	0	20.34
25	10	62	28	16.49
23	46	-72	28	14.65
61	42	-80	-14	14.35
27	34	52	26	14.01
56	-54	-36	16	13.32
7	-54	30	12	11.13
8	-50	-48	28	10.95
11	-54	-58	-2	10.67
25	22	-68	30	10.09
7	42	28	24	9.71
35	-22	-80	24	9.47
13	-18	-74	28	8.79

Table 2: Recognition phase

Size(n)	MNI X	MNI Y	MNI Z	Maximum t-value
812	-20	-94	16	33.83
197	36	-84	18	16.08
67	20	-82	-16	12.9
19	-52	16	2	12.85
34	-14	-82	-24	11.99
78	42	-82	-8	11.55
51	32	-94	4	10.6
212	-34	-84	-14	10.25
116	-56	-52	26	10.2
18	46	-58	-8	9.71
48	52	-58	2	9.5
11	-14	-104	-2	9.48
8	58	-12	32	9.37
12	-60	8	12	8.74
7	-60	0	22	8.65
14	30	-82	32	8.2
9	-52	-58	-6	8.14
7	-60	-26	32	7.26

Table 3: Across-phase

Size(n)	MNI X	MNI Y	MNI Z	Maximum t-value
14	26	32	22	27.61
32	-46	-13	-23	22.6
16	-32	44	-2	14.91
21	56	8	6	10.65
7	32	26	24	8.61
10	62	-6	-22	7.68
7	6	48	-10	6.61

References

Insausti, R., Juottonen, K., Soininen, H., Insausti, A., Partanen, K., Vainio, P., … Pitkänen, A. (1998). MR volumetric analysis of the human entorhinal, perirhinal, and temporopolar cortices. *American Journal of Neuroradiology*, *19*(4), 659–671.
